# Acoustic frequency filter based on anisotropic topological phononic crystals

**DOI:** 10.1038/s41598-017-15409-2

**Published:** 2017-11-08

**Authors:** Ze-Guo Chen, Jiajun Zhao, Jun Mei, Ying Wu

**Affiliations:** 1 King Abdullah University of Science and Technology (KAUST),Division of Computer, Electrical and Mathematical Science and Engineering (CEMSE), Thuwal, 23955-6900 Saudi Arabia; 2 Department of Physics, South China University of Technology, Guangzhou, 510640 China; 3 Present Address: GOWell International LLC, Houston, Texas 77041 USA

## Abstract

We present a design of acoustic frequency filter based on a two-dimensional anisotropic phononic crystal. The anisotropic band structure exhibits either a directional or a combined (global + directional) bandgap at certain frequency regions, depending on the geometry. When the time-reversal symmetry is broken, it may introduce a topologically nontrivial bandgap. The induced nontrivial bandgap and the original directional bandgap result in various interesting wave propagation behaviors, such as frequency filter. We develop a tight-binding model to characterize the effective Hamiltonian of the system, from which the contribution of anisotropy is explicitly shown. Different from the isotropic cases, the Zeeman-type splitting is not linear and the anisotropic bandgap makes it possible to achieve anisotropic propagation characteristics along different directions and at different frequencies.

## Introduction

Topology, a mathematic concept, was introduced to physics along with the discoveries of quantum Hall effect^1–3^. In a quantum Hall insulator, there exist non-trivial bandgaps characterized by non-zero Chern numbers that give rise to robust one-way edge states. Such non-trivial bandgaps are usually attributed to the broken time-reversal (TR) symmetry, and lead to breathtaking potential applications in spintronic devices and quantum computations^4^, which has also inspired many analogues in photonic^5–12^ and phononic crystals^13–26^. While breaking TR symmetry was realized in photonic systems by introducing the gyromagnetic material, it was considered a difficult task for phononic systems until A. Alu and his colleagues introduced airflow as a TR symmetry broken perturbation in acoustics^27^. Later, acoustic Chern insulators are demonstrated in acoustic nonreciprocal circulators with the angular-momentum bias^14,18,19,28^. These progresses open avenues for the designs of new devices to control acoustic waves.

Most of the previous research focuses on the topological property of isotropic systems with global (or complete) bandgaps. Limited efforts have been devoted to anisotropic systems with *directional (or partial)* bandgaps^29^. However, anisotropy grants more degrees of freedom in manipulating wave propagation and adds more complexity in the corresponding mathematical modeling, it would be interesting to investigate the consequences of breaking certain symmetries in an anisotropic system. For example, the topology evolution of a directional bandgap when the TR symmetry is broken and the subsequent wave propagation behaviors may bring rich physics and render more applications.

Here, we explicitly study the topological properties of anisotropic systems. We find that a two-dimensional (2D) anisotropic phononic crystal, under broken TR symmetry, possessing a combined topologically nontrivial *global* and a *directional* bandgap. The global bandgap is attributed to the broken TR symmetry, while the directional bandgap is a signature of the anisotropy. The combined bandgap enables the frequency filter functionality of the phononic crystal: a particular boundary either supports a topologically protected edge state or prohibits wave propagation, depending on the working frequency. Such phononic crystal offers a platform to engineer the topology through multiple parameters including TRS broken perturbation, geometric parameter, direction and frequency. The TRS broken perturbation is contributed by the external applied air flow, and without that, the system exhibit various topologically trivial bandgaps, *global*, *directional*, or combined, depending on geometric parameters. By applying the gradually increased external air flow, the system may experience topological transitions from a conductor or a normal insulator to a Chern insulator. We further consider the contribution of the anisotropy and find that along a certain direction, the bandgap topology is associated with the frequency. To characterize the phase transitions and capture the physical essence of anisotropy, we develop an effective Hamiltonian and classify the topological properties. Potential applications are discussed as well.

## Results

### Theoretic Model

**Figure 1:**
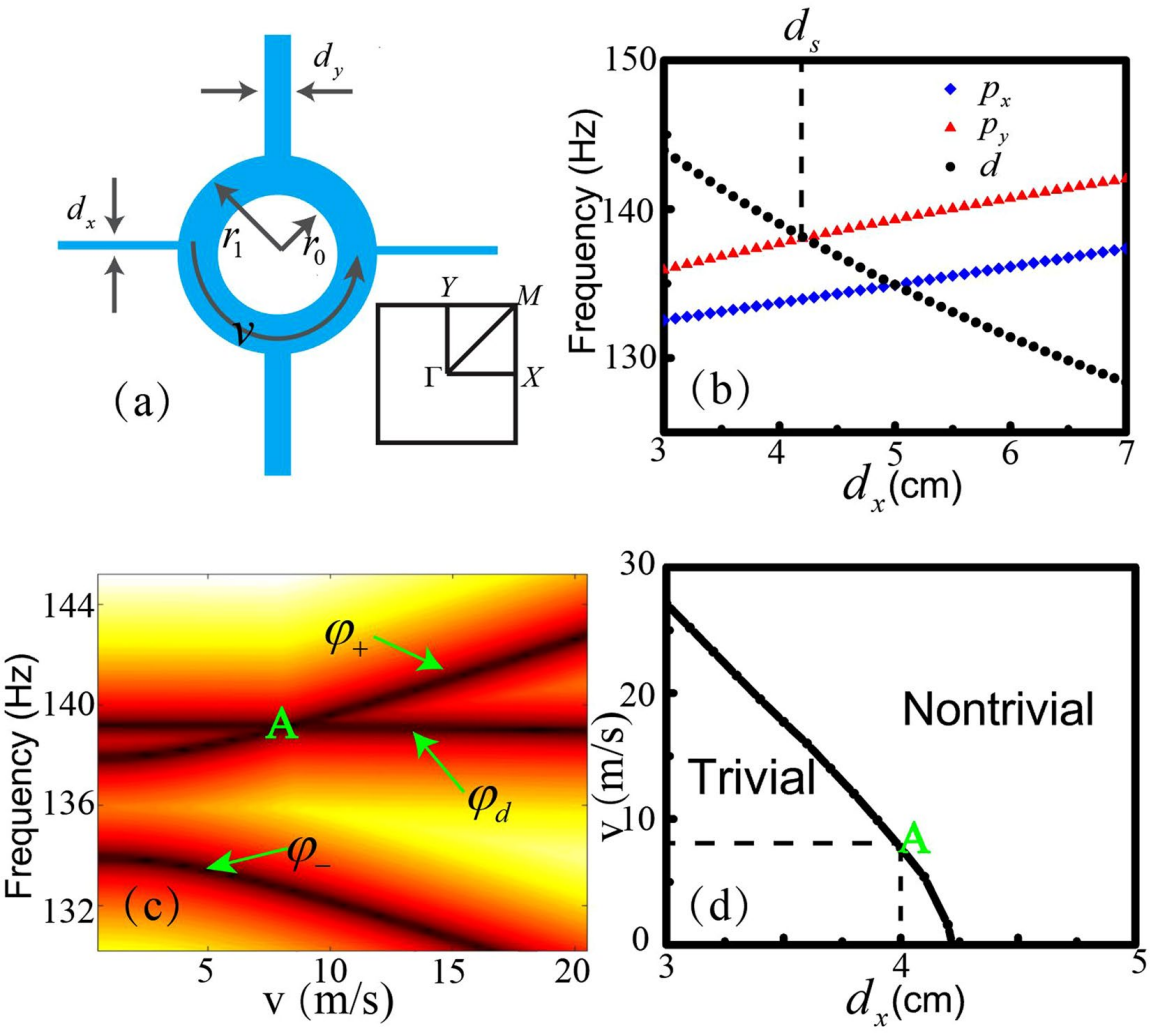
(**a**) Schematic of a unit cell of the phononic crystal. The inner and out radii of the ring ${r}_{0}=0.35\,m$, ${r}_{1}=0.5\,m$ are fixed. *d*_*x*_ and $v$ are tunable. The lower inset shows the first Brillouin Zone. (**b**) The eigenfrequencies of three interested eigenstates varies as functions of *d*_*x*_ without airflow. The black and red (blue) curves correspond to the eigenfrequency of *φ*_*d*_ and *φ*_*py*_ (*φ*_*px*_) eigenstates, respectively. *d*_*s*_ is a transition point where the global bandgap disappears and induces a semi-Dirac point. (**c**) The eigenfrequency of ${\phi }_{d}$ and ${\phi }_{+}$ $({\phi }_{-})$ versus the velocity field of the induced airflow at ${d}_{x}=0.04\,m$. The letter “A” indicates a topological transition point. (**d**) The phase diagram shows a topological transition under combined modulations of the width of the waveguide and the intensity of the airflow.

The two-dimensional anisotropic phononic crystal considered here is composed of a square array of acoustic waveguides. As illustrated in Fig. 1(a), the unit cell with lattice constant $a=2\,m$ contains a hollow ring with inner and outer radii ${r}_{0}=0.35\,m$ and ${r}_{1}=0.5\,m$, respectively, connected by four rectangular waveguides. While the lengths of these waveguides are identical, the widths of them are different, giving rise to anisotropic coupling along different directions between neighboring units. We set the width as ${d}_{y}=\kappa {d}_{x}$, where *d*_*x*_ (*d*_*y*_) indicates the widths of the horizontal (vertical) waveguides and is tunable. For simplicity but without loss of generality, the ratio of anisotropy *κ* is fixed to be 2.5. Inside the ring, the air flows counterclockwise with a velocity field distribution $V=v{\vec{e}}_{\theta }$, where ${\vec{e}}_{\theta }$ denotes the azimuthal unit vector. The acoustic wave propagation obeys the irrotational aero-acoustics equation^30^.

**Figure 2:**
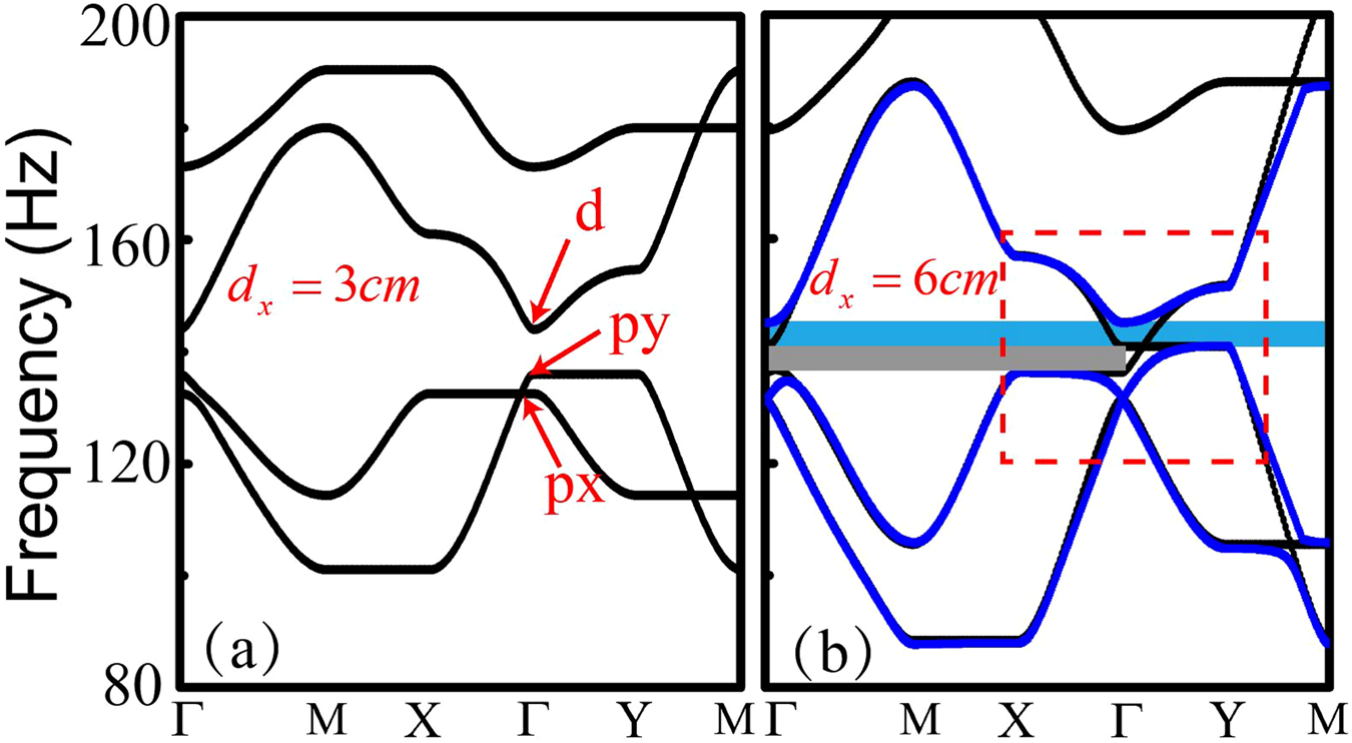
Band structure of the anisotropic phononic crystal. (**a**) ${d}_{x}=0.03\,m$ (**b**) ${d}_{x}=0.06\,m$ without airflow (black line) and with airflow $v=20\,m/s$ (blue line). The dashed box indicates the interested frequency region. The blue area indicates a global bandgap and the gray area indicates a directional bandgap.

The band structure of the phononic crystal with ${d}_{x}=0.03\,m$ without airflow is shown in Fig. 2(a), which exhibits three states at the Brillouin zone center. Their field patterns possess symmetries denoted as *d*, *px*, *py*, a convention used in classifying electron orbitals^31^. The eigenfrequencies of these states depend on the size of the rectangular waveguides. Such band structure may be modeled by the tight-binding approximation and the corresponding onsite energy of states ${\varphi }_{d}$, ${\varphi }_{px}$, ${\varphi }_{py}$ (in free space) are ${\varepsilon }_{d}$, ${\varepsilon }_{px}$, ${\varepsilon }_{py}$, respectively. When the airflow is introduced, the effective Hamiltonian, under the basis of $({\varphi }_{d},{\varphi }_{+},{\varphi }_{-})$ with ${\varphi }_{\pm }=({\varphi }_{px}\pm i{\varphi }_{py})/\sqrt{2}$, is written as:

1 $$H=[\begin{array}{ccc}{E}_{d} & \sqrt{2}{t}_{dpx}^{x}\,\sin ({k}_{x})-i\sqrt{2}\,{t}_{dpy}^{y}\,\sin ({k}_{y}) & -\sqrt{2}\,{t}_{dpx}^{x}\,\sin ({k}_{x})-i\sqrt{2}{t}_{dpy}^{y}\,\sin ({k}_{y})\\ \sqrt{2}{t}_{dpx}^{x}\,\sin ({k}_{x})+i\sqrt{2}\,{t}_{dpy}^{y}\,\sin ({k}_{y}) & -{\rm{\Delta }}z+({E}_{px}+{E}_{py})/2 & (-{E}_{px}+{E}_{py})/2\\ -\sqrt{2}\,{t}_{dpx}^{x}\,\sin ({k}_{x})+i\sqrt{2}\,{t}_{dpy}^{y}\,\sin ({k}_{y}) & (-{E}_{px}+{E}_{py})/2 & {\rm{\Delta }}z+({E}_{px}+{E}_{py})/2\end{array}],$$

where ${t}_{ij}^{l}$ (*l* = *x*, *y* represents the *x* or *y* directions, *i*, *j* indicates the orbital *d*, *px*, *py*) is the coupling coefficient of two states *ϕ*_*i*_ and *ϕ*_*j*_ between two neighboring rings and ${E}_{i}={\varepsilon }_{i}+2{t}_{ii}^{x}\,\cos ({k}_{x})+2{t}_{ii}^{y}\,\cos ({k}_{y})$. ${\rm{\Delta }}z$ represents a perturbation, induced by the airflow, that breaks TR symmetry and is proportional to the strength of the airflow *ν*. At the Γ point $({k}_{x}={k}_{y}=0)$, the eigenvalues of the effective Hamiltonian are *E*_*d*_(0), $({E}_{px}(0)+{E}_{py}(0))/2-\sqrt{{\rm{\Delta }}{z}^{2}+{f}^{2}(t)}$ and $({E}_{px}(0)+{E}_{py}(0))/2+\sqrt{{\rm{\Delta }}{z}^{2}+{f}^{2}(t)}$, respectively, where the function $f(t)=({t}_{pxpx}^{x}+{t}_{pypy}^{y})(1-\kappa )$ vanishes when the system is isotropic, i.e., $\kappa =1$.

### Topological phase transition

In the frequency region around 140 *Hz*, only fundamental mode is propagating in each narrow rectangular waveguide, making the coupling coefficients and the eigenfrequencies almost proportional to the width of the waveguides when there is no airflow, as shown in Fig. 1(b). When the airflow is applied, which can be viewed as a Zeeman-type perturbation^27^ characterized by the ${\rm{\Delta }}z$ term in Eq. (1), the eigenfrequencies of ${\phi }_{+}$, ${\phi }_{-}$ and ${\phi }_{d}$ as functions of the flow strength are plotted in Fig. 1(c). Here, ${\phi }_{+}$, ${\phi }_{-}$ and ${\phi }_{d}$ denote the Bloch states at the Brillouin zone center, which are different from the free space states mentioned earlier.. The intersecting point A in Fig. 1(c) indicates the band inversion between branches associated with ${\phi }_{+}$ and ${\phi }_{d}$ states. Such inversion, according to the Haldane model^32^, reveals the occurrence of a topological transition. For instance, when ${d}_{x}=0.04\,m$ (the case shown in Fig. 1(c)), the system is a trivial insulator for $v < 7.7\,m/s$, and a Chern insulator for larger $v$. Because eigenfrequencies of states ${\phi }_{+}$ and ${\phi }_{d}$ depend on $v$, ${d}_{x}$, and $\kappa$, the transition points at a fixed ratio of anisotropy $\kappa$ form a curve in the phase diagram displayed in Fig. 1(d).

The phase diagram suggests that the band topology can be tuned by changing widths of the rectangular waveguides and/or the velocity of the airflow. When there is no airflow, the system exhibits a *global* bandgap when *d*_*x*_ is small, as shown in Fig. 2(a). The global bandgap gradually closes as *d*_*x*_ increases towards a critical value ${d}_{s}=0.041\,m$, where the accidental degeneracy of state ${\phi }_{d}$ and ${\phi }_{py}$ occurs, and induces a semi-Dirac point^33^. Further increasing *d*_*x*_ opens a *directional* bandgap along the Γ*X* direction, highlighted in gray in Fig. 2(b).

### Frequency filter in anisotropic system

**Figure 3:**
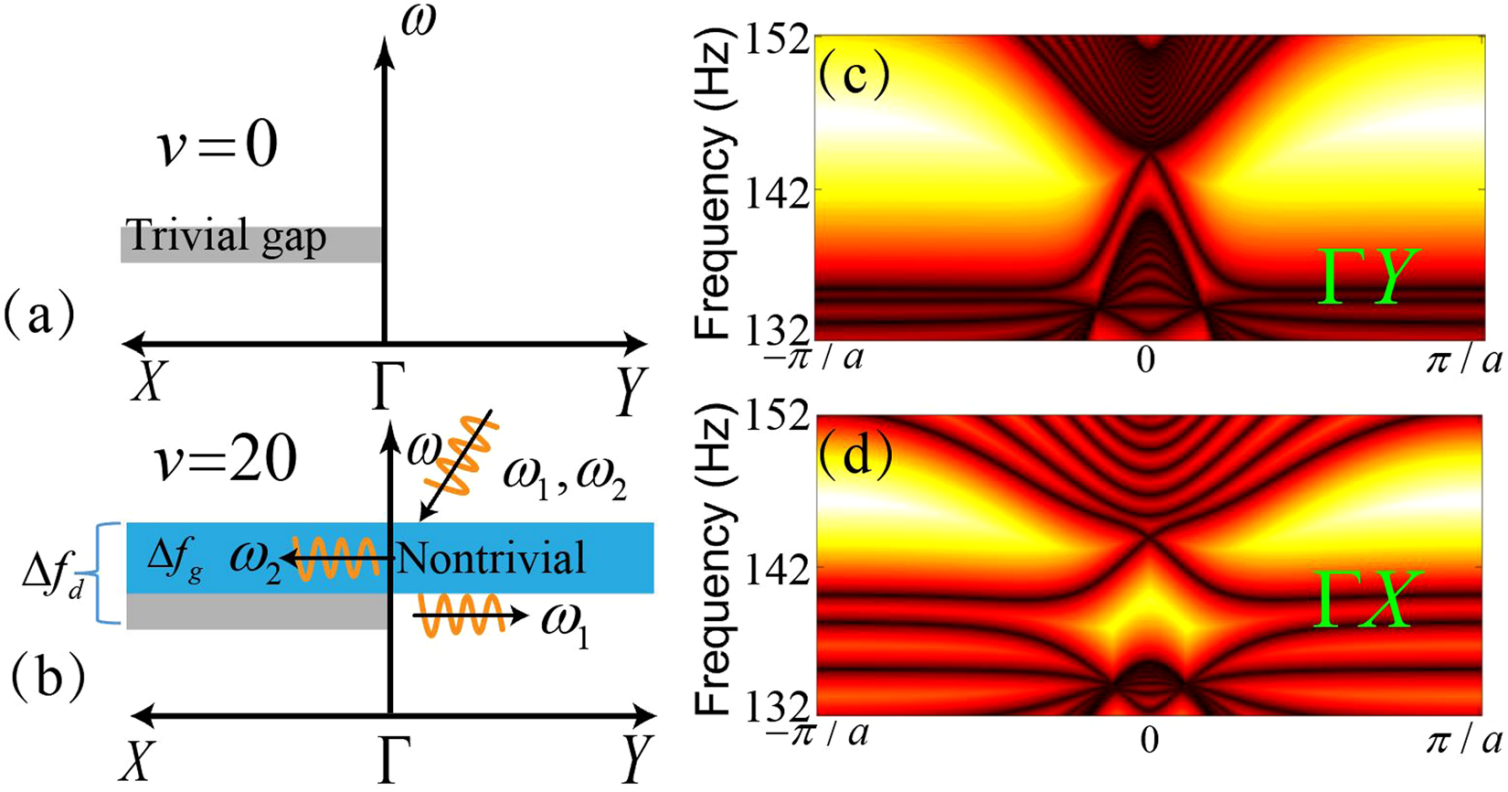
(**a**) Schematic of wave propagation behavior at ${d}_{x}=0.06\,m$ without applied airflow in a sample with boundary along Γ*X* direction. A directional bandgap along Γ*X* direction is denoted in gray. (**b**) The same as (**a**) but with $v=20\,m/s$. A global topological nontrivial bandgap is highlighted in blue. For an incident wave at frequency ${\omega }_{1}$ or ${\omega }_{2}$, it will either excite one-way propagation edge states along the Γ*X* direction (for ${\omega }_{1}$), or bulk wave propagation along the Γ*Y* direction (for ${\omega }_{2}$). (**c**,**d**) Band structures for supercells with 1 × 16 units with $v=20\,m/s$. The supercells are periodic along (**c**) Γ*Y* direction, and (**d**) Γ*X* direction, respectively. Gapless edge states are clearly shown in (**c**) while for (**d**) the edge state is gapped. The gap region corresponds to the gray area shown in (**b**).

Such a directional bandgap means that the wave propagation is forbidden along the Γ*X* direction while it is allowed along the other directions. This property is summarized schematically in Fig. 3(a). It is not clear about the changes to this directional bandgap when the TR symmetry is broken. In the following, we consider an example of an anisotropic phononic crystal with ${d}_{x}=0.06\,m$ and $v=20\,m/s$. Its band structure is plotted in Fig. 2(b) in blue curves, which shows the induced air flow opens a global bandgap and the original directional bandgap almost remains unchanged. A schematic of the band diagram of this system is illustrated in Fig. 3(b), where the global bandgap with a bandwidth ${\rm{\Delta }}{f}_{g}$ is marked in blue. From the Eq. (1), we can prove that this global bandgap is topologically nontrivial with a nonzero Chern number *C* = 1. Figure 3(b) also shows that the gap size along the Γ*X* direction is ${\rm{\Delta }}{f}_{d}$, which is larger than ${\rm{\Delta }}{f}_{g}$, meaning that the gap along the Γ*X* direction would exhibit a *mixed* behavior that cannot be simply defined as trivial or nontrivial. To examine the topological property of this anisotropic phononic crystal, we study the edge states. It is well known that for a system possessing a topologically nontrivial bandgap, there exists a gapless edge state at its interface with a trivial insulator. We calculate the band structures of two different supercells consisting of 1 × 16 unit cells. The first one is infinite along the Γ*Y* direction and terminated by rigid boundaries along the Γ*X* direction. The gapless edge state is clearly shown in Fig. 3(c). The second one is infinite along the Γ*X* direction. In this case, the edge state only exists within the frequency region ${\rm{\Delta }}{f}_{g}$ $(\mathrm{140}{\rm{.7}}\,Hz-145.2\,Hz)$, and below the edge state there is a bandgap covering a frequency range ${\rm{136.1}}\,Hz-140.7\,Hz$ with gap width ${\rm{\Delta }}{f}_{d}-{\rm{\Delta }}{f}_{g}$, as shown in Fig. 3(d). Therefore, for a sample with a boundary along the Γ*X* direction, it will “select” the type of propagating waves according to the frequency. As illustrated schematically in Fig. 3(b), the sample supports one-way propagation edge state along the Γ*X* direction at ${\omega }_{2}$, while a bulk state propagating along the Γ*Y* direction is supported at ${\omega }_{1}$.

## Discussions and Conclusions

**Figure 4:**
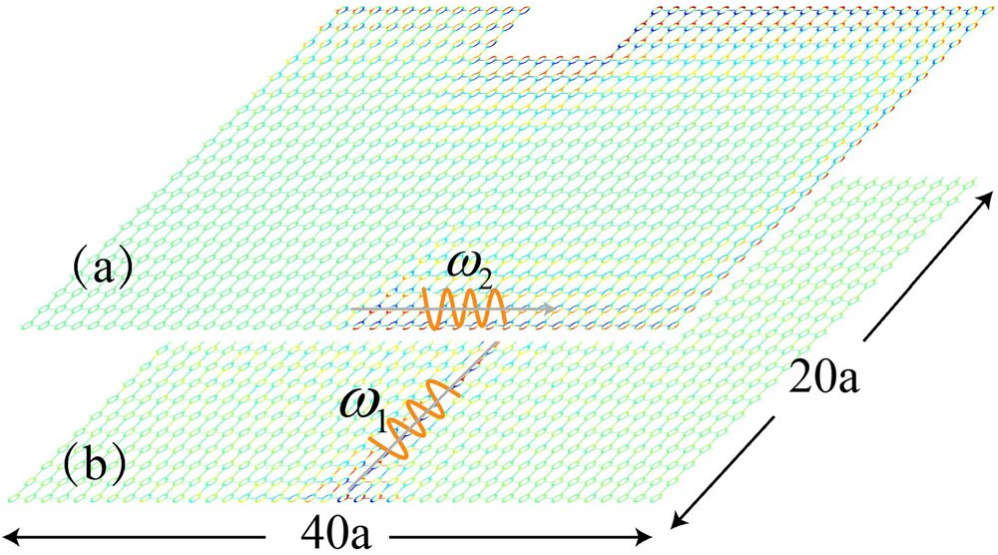
Chiral edge state and directional wave propagation. (**a**) Acoustic Chern insulator when excited by a source at frequency ${\omega }_{2}=144\,Hz$. One-way edge state propagates counter-clockwise. (**b**) Directional wave propagation (bulk wave) when excited by a source at frequency ${\omega }_{1}=139\,Hz$. No wave is seen on the boundary.

To verify our predictions of the frequency-dependent propagating behavior, we perform finite-element simulations of a finite-sized sample. It contains 20 × 40 unit cells. We impose a source, with two different frequencies ${\omega }_{1}$ and ${\omega }_{2}$, at the bottom of the sample. At ${\omega }_{2}$, the system behaves as a Chern insulator with a topological protected edge state propagating at the boundary as shown in Fig. 4(a), which resembles the property in an isotropic acoustic Chern insulator. However, the strikingly difference occurs at ${\omega }_{1}$. In the isotropic case, ${\omega }_{1}$ corresponds to bulk state frequency, and the wave propagation is supported everywhere including the boundary^28^. While in our anisotropic Chern insulator, the system possesses a directional bandgap that forbids wave propagation along the Γ*X* direction at ${\omega }_{1}$, the wave cannot propagate along the boundary as manifested in Fig. 4(b). Thus, the boundary can be viewed as a frequency filter.

In conclusion, we report our design of an anisotropic topological phononic crystal which can work as a frequency filter. It exhibits a tunable topological transition point as well as a tunable *directional* bandgap. The combined topological nontrivial *global* bandgap and the directional bandgap is systematically studied by using a tight-binding model and numerical simulations. We find the wave propagation behavior at a particular boundary depends on the frequency, and we demonstrate the functionality of a frequency filter: at certain frequencies, the boundary allows one-way propagation edge state, while at other frequencies, it forbids wave propagation. The mechanism is universal and would not be limited to acoustics. We believe our findings can inspire more designs and applications based on topological insulators.

## Methods

Throughout the paper, the Finite Element Method (FEM) based on commercial software COMSOL Multiphysics is employed for the band structure computations and the simulations. Plane wave radiation boundary conditions are set on the outer boundaries of simulation domain. The largest mesh element size is set smaller than 1/20 of the shortest wavelength. The topological invariant is calculated based on Eq. (1).

## References

[1] Hasan MZ, Kane CL (2010). Topological insulators. Rev. Mod. Phys..

[2] Moore JE (2010). The birth of topological insulators. Nature.

[3] Qi XL, Zhang SC (2011). Topological insulators and superconductors. Rev. Mod. Phys..

[4] Nayak C, Simon SH, Stern A, Freedman M, Das Sarma S (2008). Non-Abelian anyons and topological quantum computation. Rev. Mod. Phys..

[5] Haldane FDM, Raghu S (2008). Possible Realization of Directional Optical Waveguides in Photonic Crystals with Broken Time-Reversal Symmetry. Phys. Rev. Lett..

[6] Wang Z, Chong YD, Joannopoulos JD, Soljačić M (2008). Reflection-Free One-Way Edge Modes in a Gyromagnetic Photonic Crystal. Phys. Rev. Lett..

[7] Khanikaev AB (2013). Photonic topological insulators. Nat. Mater..

[8] Lu L, Joannopoulos JD, Soljacic M (2014). Topological photonics. Nat. Photon..

[9] Ma T, Khanikaev AB, Mousavi SH, Shvets G (2015). Guiding Electromagnetic Waves around Sharp Corners: Topologically Protected Photonic Transport in Metawaveguides. Phys. Rev. Lett..

[10] Wu L-H, Hu X (2015). Scheme for Achieving a Topological Photonic Crystal by Using Dielectric Material. Phys. Rev. Lett..

[11] He C (2016). Photonic topological insulator with broken time-reversal symmetry. Proc. Natl. Acad. Sci. USA.

[12] Peano V, Brendel C, Schmidt M, Marquardt F (2015). Topological Phases of Sound and Light. Phys. Rev. X.

[13] Mousavi SH, Khanikaev AB, Wang Z (2015). Topologically protected elastic waves in phononic metamaterials. Nat. Commun..

[14] Ni X (2015). Topologically protected one-way edge mode in networks of acoustic resonators with circulating air flow. New J. Phys..

[15] Süsstrunk R, Huber SD (2015). Observation of phononic helical edge states in a mechanical topological insulator. Science.

[16] Wang P, Lu L, Bertoldi K (2015). Topological Phononic Crystals with One-Way Elastic Edge Waves. Phys. Rev. Lett..

[17] Xiao M (2015). Geometric phase and band inversion in periodic acoustic systems. Nat. Phys..

[18] Yang Z (2015). Topological Acoustics. Phys. Rev. Lett..

[19] Chen Z-G, Wu Y (2016). Tunable Topological Phononic Crystals. Phys. Rev. Applied.

[20] He C (2016). Acoustic topological insulator and robust one-way sound transport. Nat. Phys..

[21] Zhang Z (2017). Topological Creation of Acoustic Pseudospin Multipoles in a Flow-Free Symmetry-Broken Metamaterial Lattice. Phys. Rev. Lett..

[22] Mei J, Chen Z, Wu Y (2016). Pseudo-time-reversal symmetry and topological edge states in two-dimensional acoustic crystals. Sci. Rep.

[23] Swinteck N (2015). Bulk elastic waves with unidirectional backscattering-immune topological states in a time-dependent superlattice. J. Appl. Phys..

[24] Brendel C, Peano V, Painter OJ, Marquardt F (2017). Pseudomagnetic fields for sound at the nanoscale. Proc. Natl. Acad. Sci. USA.

[25] Prodan E, Prodan C (2009). Topological Phonon Modes and Their Role in Dynamic Instability of Microtubules. Phys. Rev. Lett..

[26] Lu J (2017). Observation of topological valley transport of sound in sonic crystals. Nat. Phys..

[27] Fleury R, Sounas DL, Sieck CF, Haberman MR, Alù A (2014). Sound Isolation and Giant Linear Nonreciprocity in a Compact Acoustic Circulator. Science.

[28] Khanikaev AB, Fleury R, Mousavi SH, Alu A (2015). Topologically robust sound propagation in an angular-momentum-biased graphene-like resonator lattice. Nat. Commun..

[29] He W-Y, Chan CT (2015). The Emergence of Dirac points in Photonic Crystals with Mirror Symmetry. Sci. Rep.

[30] Brekhovskikh, L. M. & Lysanov, I. U. P. *Fundamentals of Ocean Acoustics*. (Springer-Verlag New York, 2003).

[31] Sakoda, K. *Optical Properties of Photonic Crystals*. (Springer Berlin Heidelberg, 2005).

[32] Haldane FDM (1988). Model for a Quantum Hall Effect without Landau Levels: Condensed-Matter Realization of the “Parity Anomaly”. Phys. Rev. Lett..

[33] Wu Y (2014). A semi-Dirac point and an electromagnetic topological transition in a dielectric photonic crystal. Opt. Express.

